# Hypoglycemic Properties of the Aqueous Extract from the Stem Bark of* Ceiba pentandra* in Dexamethasone-Induced Insulin Resistant Rats

**DOI:** 10.1155/2018/4234981

**Published:** 2018-09-16

**Authors:** Christian Kuété Fofié, Elvine Pami Nguelefack-Mbuyo, Nole Tsabang, Albert Kamanyi, Télesphore Benoît Nguelefack

**Affiliations:** ^1^Laboratory of Animal Physiology and Phytopharmacology, Faculty of Science, University of Dschang, P.O. Box 67, Dschang, Cameroon; ^2^Institut de Recherche Médicale et d'Etude des Plantes Médicinales (IMPM), Cameroon

## Abstract

Parts of* Ceiba pentandra* are wildly used in Africa to treat diabetes and previous works have demonstrated their* in vivo* antidiabetic effects on type 1 diabetes models. In addition, it has been recently shown that the decoction and the methanol extract from the stem bark of* C. pentandra* potentiate* in vitro,* the peripheral glucose consumption by the liver and skeletal muscle slices. But nothing is known about its effect on type II diabetes, especially on insulin resistance condition. We investigated herein the antihyperglycemic, insulin-sensitizing potential, and cardioprotective effects of the dried decoction from the stem bark of* Ceiba pentandra* (DCP) in dexamethasone-induced insulin resistant rats. DCP phytochemical analysis using LC-MS showed the presence of many compounds, including 8-formyl-7-hydroxy-5-isopropyl-2-methoxy-3-methyl-1,4-naphthaquinone, 2,4,6-trimethoxyphenol, and vavain. Wistar rats were given intramuscularly (*i.m.*) dexamethasone (1 mg/kg/day) alone or concomitantly with oral doses of DCP (75 or 150 mg/kg/day) or metformin (40 mg/kg/day) for 9 days. Parameters such as body weight, glycemia, oral glucose tolerance, plasma triglycerides and cholesterol, blood pressure, and heart rate were evaluated. Moreover, cardiac, hepatic and aortic antioxidants (reduced glutathione, catalase, and superoxide dismutase), malondialdehyde level, and nitric oxide content were determined. DCP decreased glycemia by up to 34% and corrected the impairment of glucose tolerance induced by dexamethasone but has no significant effect on blood pressure and heart rate. DCP reduced the total plasma cholesterol and triglycerides as compared to animals treated only with dexamethasone. DCP also increased catalase, glutathione, and NO levels impaired by dexamethasone, without any effect on SOD and malondialdehyde. In conclusion, the decoction of the stem bark of* Ceiba pentandra* has insulin sensitive effects as demonstrated by the improvement of glucose tolerance, oxidative status, and plasma lipid profile. This extract may therefore be a good candidate for the treatment of type II diabetes.

## 1. Introduction

Diabetes is the most frequently encountered metabolic disease in our societies today. Its prevalence has increased dramatically in recent years and it is considered by WHO as a serious public health problem. According to the IDF estimation, nearly 451 million people were suffering from diabetes in 2017 worldwide [1]. This figure is said to reach 693 million by 2045 if the current growth rate continues [1]. According to Zheng et al. [2], about 90% of diabetic patients suffer from type 2 diabetes mellitus (T2DM), which is a complex heterogeneous metabolic condition characterized by chronic hyperglycemia associated with insulin impairment, specific organic complications, and cardiovascular diseases (CVD). CVD is the most prevalent cause of mortality and morbidity in diabetic population [3]. Evidence implicates hyperglycemia-derived oxygen free radicals as mediators of diabetic complications [4]. These include increased polyol pathway flux, advanced glycation end products formation, hexosamine pathway flux, and activation of protein kinase C.

In general, because of the complexity of the disease and the specificity of conventional antidiabetic drugs, multidrug therapy is often required in order to achieve an effective relief in some patients. Unfortunately, multidrug therapy overlaps inherent side effects [5] and increases therefore the risk of drug intoxication. These limits give the rationale for the assessment of new methods that are both less toxic and more effective. Phytotherapy is one of the most interesting means to achieve this goal.

*Ceiba pentandra* (Bombacaceae), commonly called the silk-cotton tree, is a highly coveted plant in the African traditional medicine, because of its large medicinal properties. The bark and the leaves of this tropical tree are used against diabetes, dizziness, headache, hypertension, and fever. Indeed, many authors showed that the decoction or the methylene/chloride extract from the root, stem bark, or leaves of* C. pentandra* tree have antihyperglycemic effect or antidiabetic effect [6–8]. Our previous studies showed that the aqueous (decoction and maceration) and the methanol extracts from the stem bark of* Ceiba pentandra* potentiated peripheral glucose consumption, reduced liver glucose release, and possessed antioxidant properties, with the decoction being the most efficient [9]. Despite these interesting pharmacological activities, no study has reported the effects of* Ceiba pentandra* on glucose metabolism and cardiovascular complications in an insulin resistance condition. Many experimental models have been developed to mimic this latter condition among which the glucocorticoid (dexamethasone) model. This experimental metabolic model is already well characterized as being associated with a decrease in body weight [10]; an increase of glycemia and insulinemia [11, 12] which mark the presence of an insulin resistance; a loss in muscle mass associated with liver hypertrophy [10]; an alteration of protein [13]; and lipid profiles [14]. Besides metabolic changes, some cardiovascular alterations have also been described in this model such as arterial hypertension associated with an increased oxidative stress [15, 16] and cardiac hypertrophy [17].

Therefore, the aim of this work was to investigate the effect of the decoction of* Ceiba pentandra *stem bark on a glucose metabolism and impaired cardiovascular parameters in an experimental model of insulin resistance induced by dexamethasone.

## 2. Materials and Methods

### 2.1. Chemicals

Dexamethasone was obtained from Enzo Life Sciences (USA), D-glucose, metformin, urethane, and thiobarbituric acid were purchased from Fluka. Tris, sodium citrate, dithiobisnitrobenzoate, hydrogen peroxide, adrenaline, and trichloroacetic acid were purchased from Sigma-Aldrich, Germany. NaHCO_3_ and Na_2_HPO_4_ were provided by Riedel-de Haën AG. Na_2_CO_3_, KH_2_PO_4_, NaCl, and orthophosphoric acid were purchased from BDH (Chemicals Ltd., Poole, England). Naphtylethylene diamine and acetic acid were purchased from Merck and sulfanilamide was purchased from Alfa Aesar, sodium nitrite from Analytica Reagent, and copper sulfate from Fisher, while sodium-potassium tartrate, potassium iodine, sodium hydroxide, and potassium dichromate were obtained from Carl Roth (Germany). Cholesterol and triglycerides kits were purchased at IMMESCO (Italy).

### 2.2. Plant Material and Preparation of the Extract

The leaves, stem bark, and flowers of* Ceiba pentandra* were collected in Yaoundé (Center Region, Cameroon) between January and February by Dr. Tsabang Nolé. The plant materials were taken to the National Herbarium in Yaoundé, where the authentication was done by comparing the samples to the specimen number HNC 43623. The fresh stem barks were air-dried and ground into a powder. Two hundred grams of this powder was then boiled in 1.5 l of distilled water for 20 minutes. After filtration, the filtrate was freeze-dried to obtain 8.86 g of aqueous extract pellet, corresponding to an extraction yield of 4.41%.

### 2.3. Animal

Both adult male and female Wistar rats of 280-320 g weight and aged 5 to 6 months were randomly selected from our local colonies. They were raised in the animal house of the Faculty of Science, University of Dschang, Cameroon. The animals were treated in accordance with the internationally accepted standard ethical guidelines for laboratory animal use and care as described in the European Community Guidelines [18]. Throughout the experimental period, the animals received standard rat diet and tap water* ad libitum*.

### 2.4. LC/MS

DCP was subjected to LC-MS analysis. The following parameters were used for experiments: spray voltage of 4.5 kV and capillary temperature of 200°C. Nitrogen was used as sheath gas (10 l/min). The spectrometer was attached to an Ultimate 3000 (Thermo Fisher, USA) UHPLC system consisting of LC-pump, Diode Array Detector (DAD) (*λ*: 190-600 nm), autosampler (injection volume 10 *μ*l), and column oven (40°C). The separations were performed using a Synergi MAX-RP 100A (50x2 mm, 2.5*μ*m particle size) with a H_2_O (+0.1% HCOOH) (A)/acetonitrile (+0.1% HCOOH) (B) gradient (flow rate 500 *μ*L/min, injection volume 10 *μ*l). Samples were analyzed using a gradient program as follows: 95% A isocratic for 1.5 min and linear gradient to 100% B over 6 min, and, after 100% B isocratic for 2 min, the system returned to its initial condition (90% A) within 1 min and was equilibrated for 1 min. High resolution mass spectra were obtained with a QTOF Spectrometer (Bruker, Germany) equipped with a HESI source. The spectrometer was operated in positive mode (mass range: 100-1500, with a scan rate of 1.00 Hz) with automatic gain control to provide high-accuracy mass measurements within 0.40 ppm deviation using Na Formate as calibrant.

### 2.5. Induction of Insulin Resistance and Experimental Protocol

Induction of insulin resistance by dexamethasone was performed according to the protocol previously described [19] with some modifications. Animals were divided into 5 groups of six rats each (3 males and 3 females). Rats in the first group served as normal control and received per os (*p.o*) distilled water (10 ml/kg/day) and intramuscular (i.m.) injection of NaCl 0.9% (1 ml/kg/day). Group 2 considered as insulin resistant control received daily intramuscular injection of dexamethasone (1 mg/kg/day) and distilled water (10 ml /kg/day,* p.o.*). Rats in experimental groups 3 and 4 were treated orally with the decoction of* Ceiba pentandra *at respective doses of 75 (DCP 75) and 150 mg/kg/day (DCP 150) plus daily injection of dexamethasone, while rats in group 5 (MET 40) were treated with standard drug metformin (40 mg/kg/day,* p.o*.) and dexamethasone. Each animal received his respective assigned treatment for a period of 8 days. The dose of dexamethasone (1 mg/kg/day) and metformin (40 mg/kg/day) was selected based on previous studies [20, 21].

### 2.6. Measurement of Blood Glucose and Oral Glucose Tolerance Test

Fasting blood glucose level was measured in tail blood samples. After a 6-h fasting on day 0 (before treatment) and day 9 (before the treatment of the day), basal glycemia was determined with a glucose analyzer (glucometer Accuk-Check). An oral glucose tolerance test (OGTT) was performed after the basal glycemia measurement of day nine. For this purpose, each animal received orally 2.5 g/kg of glucose and their glycemia was further determined at 30, 60, 90, and 120 min after glucose load.

### 2.7. Blood Pressure and Heart Rate Recording

On day 10, blood pressure and heart rate were determined using a standard invasive method as described previously [22]. Briefly, animals were anesthetized by intraperitoneal administration of urethane at the dose of 1.5 g/kg and a catheter filled with Mac-even heparinized solution was inserted into the left carotid artery. The catheter was connected to a blood pressure transducer model Ugo Basile PRC 21k-10 coupled to an Ugo Basile Unirecord model 7050 for direct blood pressure measurement. A stabilization period of 30 minutes was observed before any recording. Heart rate was determined using pulse intervals.

### 2.8. Blood and Organ Sample Collection

Immediately after recording of blood pressure and heart rate, blood samples were collected from the abdominal artery and centrifuged at 3000 rpm for 10 minutes. The plasma obtained was stored at −20°C for lipid assay. The liver, the heart, and the thoracic aorta were quickly removed and weighed. Thereafter, the left ventricular was separated from the heart and weighed. The left ventricular index was calculated using the following formula.

Left ventricular index (%) = (Left ventricle mass/Heart mass) × 100. The collected organs were crushed in Tris-buffer (pH, 7.4; 10 mM), centrifuged (TGL-16M, Loncare centrifuge) at 10.000 rpm for 15 minutes at 4°C and the supernatant was used to assay tissue nitric oxide, superoxide dismutase (SOD), catalase (CAT), glutathione (GSH), malondialdehyde (MDA), and protein content.

### 2.9. Biochemical Analysis

Total plasma cholesterol and triglycerides levels were measured spectrophotometrically using commercial kits and according to the manufacturers' protocols. Both tissue proteins and nitric oxide (NO) content were estimated by the method of Biuret [23] and Giustarini et al. [24], respectively. The beneficial effect of the plant extract on oxidative stress was determined by assaying enzymatic and nonenzymatic antioxidant status. MDA level was determined by the method of Olszewska-Słonina et al. [25], GSH content as described by Giustarini et al. [26], SOD activity by the method of Serra et al. [27], and catalase activity as reported by Hadwan [28].

### 2.10. Statistical Analysis

Results are expressed as mean ± SEM. Data were analyzed using one-way and two-way ANOVA, followed, respectively, by Tukey's and Bonferroni's posttest. Student's* t*-test was used for intragroup comparison of the glycemia of days 0 and 9. All the analyses were performed with GraphPad Prism 5.01 software package. P-value less than 0.05 was considered as statistically significant.

## 3. Results

### 3.1. Phytochemical Analysis

**Figure 1:**
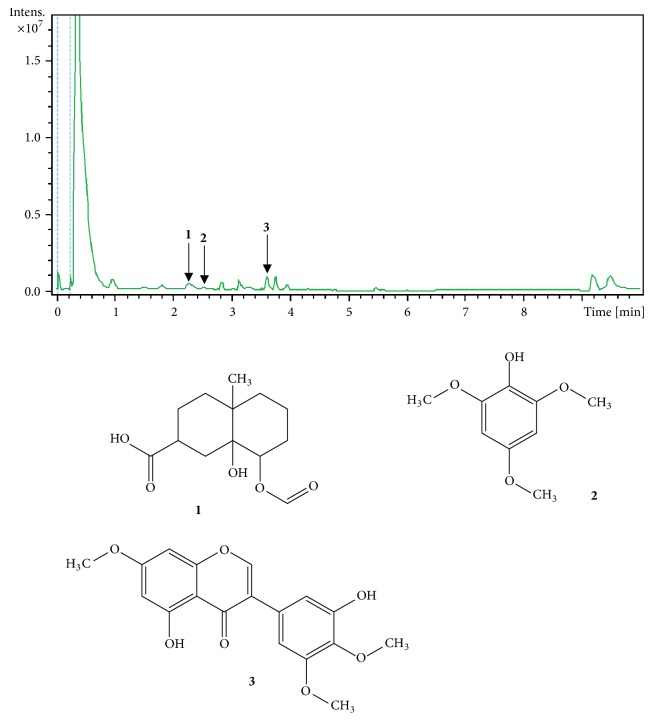
LC fingerprint of the decoction of the stem bark of* Ceiba pentandra*, detected with UV 190-600 nm. Identified compounds (1-3) are indicated by peak numbers on the chromatogram. 1: 8-(formyloxy)-8a-hydroxy-4a-methyldecahydro-2-naphthalenecarboxylic acid; 2: 2,4,6-Trimethoxyphenol; 3: 5,3′-dihydroxy-7,4′,5′-trimethoxyisoflavone or vavain.

LC-MS was used to determine DCP profile shown in Figure 1. The combination of data from the literature and information from the MS spectral allows tentative identification of three compounds (peaks numbered 1-3). Compound** 1** appears at RT 2.3 min with [M+H]+ at m/z 257 and was identified as 8-(formyloxy)-8a-hydroxy-4a-methyldecahydro-2-naphthalene carboxylic acid [29, 30]. Compound** 2 **(RT 2.6 min) showed [M+H]+ at m/z 185 and was identified as 2,4,6-trimethoxyphenol [31, 32]. Compound** 3 **showed peak at 3.6 min, an [M+H] + at* m/z *345 and was identified as 5,3′-dihydroxy-7,4′,5′-trimethoxyisoflavone or vavain [33, 34] (Figure 1).

### 3.2. Effects of the Decoction of* Ceiba pentandra* on Fasting Glycemia

**Figure 2:**
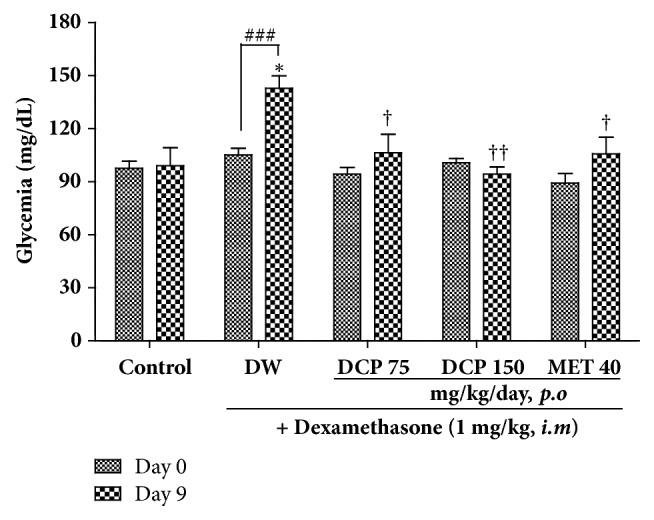
Basal glycemia and effect of* Ceiba pentandra* on the 9 days dexamethasone-induced hyperglycemia in rats. Values are expressed as mean ± SEM from 6 rats; **∗***p*<0.05 with respect to control group at day 9 (ANOVA + Tukey's posttest). ^†^*p*<0.05, ^††^*p*<0.01with respect to dexamethasone group (ANOVA + Tukey's posttest). ^###^p<0.001, with respect to the same group before treatment (Student's* t*-test). Met: metformin; DCP: decoction from the stem bark of* Ceiba pentandra.*

At the end of the 9 days of treatment, dexamethasone significantly increases animals' glycemia by 35% as compared to the value of the same group before treatment and by 44% as compared to the normal control group. Oral administration of DCP significantly reduced the hyperglycemia induced by dexamethasone in a dose-dependent manner, with the maximal effect of 33% obtained at the dose of 150 mg/kg/day. Metformin used as positive control reduced glycemia by 26% as compared to the dexamethasone group. Except the dexamethasone group, none of the treated groups showed a significant difference when comparing his glycemia before and after the treatment period (Figure 2).

### 3.3. Effect of* C. pentandra* Decoction on Oral Glucose Tolerance Test

**Figure 3:**
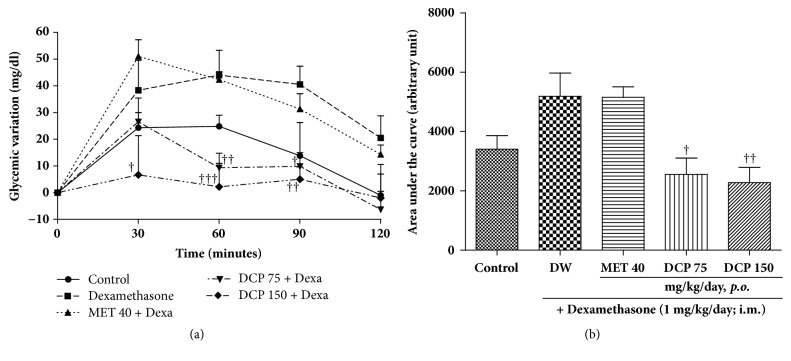
Effect of* Ceiba pentandra* on the glycemic variation (a) and AUC (b) from oral glucose tolerance test performed on day nine of treatment. Values are expressed as mean ± SEM from 6 rats; ^†^*p*<0.05, ^††^*p*<0.01, ^†††^*p*<0.001, with respect to dexamethasone (Dexa) + distilled water (DW) group. Met: metformin; DCP: decoction from the stem bark of* Ceiba pentandra*.

Results of the oral glucose tolerance test (Figure 3(a)) showed that, 30 minutes after glucose load, the difference in blood glucose of rats receiving dexamethasone alone was 57% higher than that of healthy control animals. At the same time, the difference in blood glucose of rats receiving concomitantly dexamethasone and DCP at doses of 75 and 150 mg/kg were, respectively, 31% and 83% lower, as compared to that of the dexamethasone group. In contrast to the effects induced by DCP, the difference in blood glucose in the metformin-treated animals was 25% higher than in the dexamethasone group. Two hours after glucose load, the increase in glycemia was still, respectively, 21.5 and 15.3 mg/dl higher in dexamethasone and metformin groups than in the normal control. In contrast, at the same time point, glycemia in DCP treated animals was lower than that of normal control.

The AUC plotted from the oral glucose tolerance test revealed that dexamethasone-induced impaired glucose tolerance. DCP significantly corrected this impairment by 34 and 45% at respective doses of 75 and 150 mg/kg/day. Metformin as well significantly reduced the impairment by 25% (Figure 3(b)).

### 3.4. Effects of Different Treatment on Mean Arterial Blood Pressure and Heart Rate

**Figure 4:**
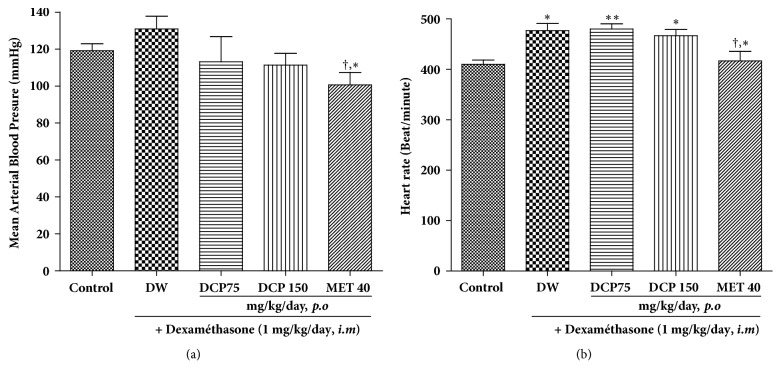
Effect of* Ceiba pentandra* on the mean arterial blood pressure (a) and the heart rate (b) of animals treated with dexamethasone. Values are expressed as mean ± SEM from 6 rats; **∗***p*<0.05; **∗∗***p*<0.01, with respect to control group. ^†^P<0.05, with respect to dexamethasone group. Met: metformin, DCP: decoction from the stem bark of* Ceiba pentandra.*

As shown in Figure 4(a), 9 days treatment with dexamethasone induced a 10% increase in arterial blood pressure but this was not statistically significant (p > 0.05) as compared to that of normal control rats. DCP treatment induced a reduction in a dose-dependent manner, with a maximal effect of 14% at the dose of 150 mg/kg/day as compared to rats treated with dexamethasone only. Repeated injection of dexamethasone significantly increased the heart rate and only metformin coadministration was able to inhibit this effect, with a significant reduction of 13% (Figure 4(b)).

### 3.5. Effects of Different Treatment on Body Weight and Organs Mass

**Figure 5:**
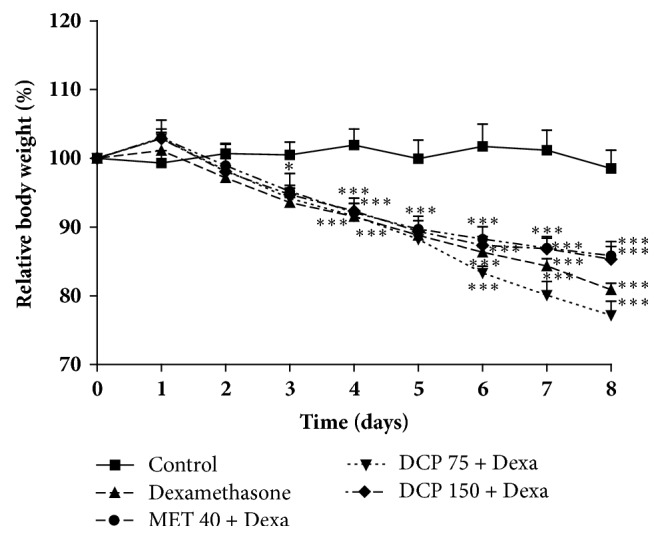
Effects of* Ceiba pentandra* on the relative body weight of animals after 9 days of treatment. Values are expressed as mean ± SEM from 6 rats; **∗***p*<0.05; **∗∗***p*<0.001, **∗∗∗**P<0.001, with respect to control group. Met: metformin, DCP: decoction from the stem bark of* Ceiba pentandra.*

Repeated dexamethasone administration induced a significant decrease of rats' body weight when compared to normal control group. Neither DCP nor metformin significantly reversed this effect when coadministered with dexamethasone (Figure 5).

**Figure 6:**
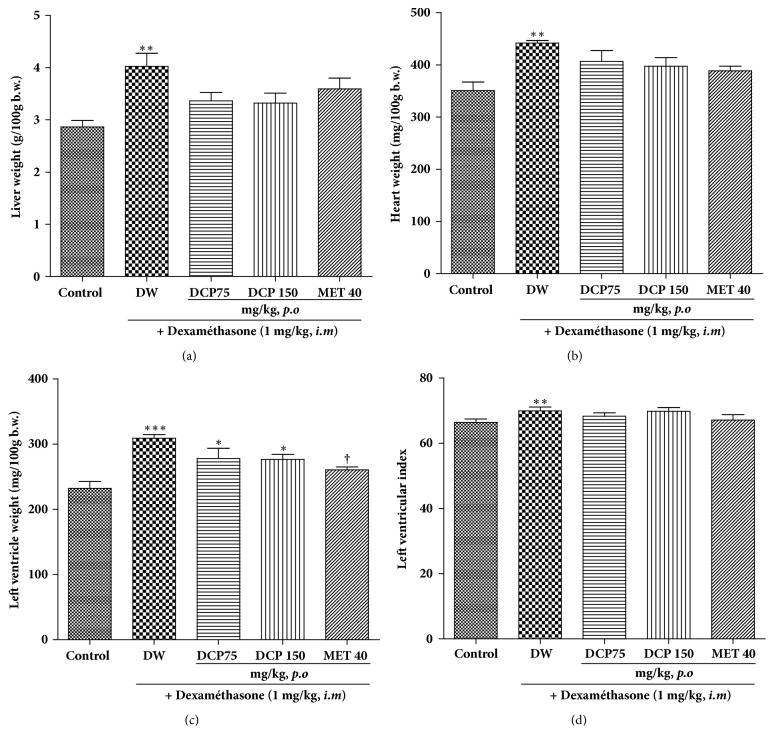
Effect of* Ceiba pentandra* on liver, heart and left ventricle relative weight in dexamethasone-treated animals. Values are expressed as mean ± SEM from 6 rats; *∗p*<0.05, *∗∗p*<0.01, *∗ ***∗∗***p*<0.001, with respect to control group and ^†^*P*<0.05, with respect to dexamethasone group. Met: metformin; DCP: decoction from the stem bark of* Ceiba pentandra.*

The relative weight of the liver and the heart was increased by 40 and 26%, respectively, in dexamethasone-treated subjects. This liver hypertrophy induced by dexamethasone was reduced by 16, 17, and 11%, respectively, by DCP75, DCP150, and metformin (Figure 6(a)). Likewise, DCP and metformin also decreased cardiac hypertrophy by 8%, 10%, and 12%, respectively (Figure 6(b)). Significant increases (p < 0.05) in the left ventricle relative weight and the left ventricular index were observed in dexamethasone-treated animals as compared to normal control group. DCP at all doses used failed to significantly reduce the ventricular hypertrophy, while metformin induced a significant reduction of 15% (Figures 6(c) and 6(d)). Nevertheless, when considering the raw organ weights, it appears that no treatment induced a significant variation excepted that the left ventricular index was significantly (p<0.05) increased by dexamethasone administration (data not shown).

### 3.6. Effect of Different Treatment on Plasma Cholesterol and Triglycerides

The plasma concentration of total cholesterol was not modified by dexamethasone administration. Nevertheless, DCP treatment led to a marked reduction of the parameter. Animals receiving DCP at the dose of 150 mg/kg/day showed a reduction of about 30%, as compared to both normal control and dexamethasone-treated animals. Dexamethasone significantly reduced the plasma concentration of HDL cholesterol but neither DCP nor metformin was able to normalize the parameter. LDL cholesterol was dose-dependently reduced by DCP, with a significant reduction of 64% observed at the dose of 150 mg/kg/day. Concerning plasmatic triglycerides, dexamethasone administration drastically increased its level by 106%. DCP at the dose of 150 mg/kg/day and metformin as well significantly reduced the hypertriglyceridemia induced by dexamethasone by 35% and 48%, respectively (Table 1).

### 3.7. Effect of Different Treatments on Tissue Proteins Content and Tissue Oxidative Stress Parameters

Dexamethasone administered alone induced a reduction in proteins content in the liver, heart, and aorta. A significant reduction was observed only in the liver as compared to normal control. In all the organs, DCP increased the protein concentration but the increase was significant only in the liver as compared to dexamethasone-treated animals (Table 2).

Except the glutathione content in the liver, repeated intramuscular administration of dexamethasone did not significantly affect the content of the marker of oxidative stress. DCP administered at the dose of 75 mg/kg/day, significantly elevated the catalase content in the liver by 26%. The same parameter was instead significantly reduced by metformin. Similarly, DCP at 75 mg/kg/day significantly increased the catalase level in the heart by 108%. None of the treatments affected this parameter in the aorta although it tends to decrease in animals treated with metformin. Also, they did not significantly affect the SOD and the MDA content in all the tissues used. Concerning GSH, dexamethasone repeated administration significantly reduced it by 46% in the liver. In the meantime, DCP (75 and 150 mg/kg/day) and metformin significantly corrected this impairment by increasing GSH content by 59%, 81%, and 63% respectively, as compared to the dexamethasone group (Table 2).

DCP induced a nonsignificant concentration-dependent increase in the NO content in the liver. Metformin significantly increased the NO concentration as compared to both normal control (125%) and dexamethasone-treated animals (176%). The same parameter was also increased in the heart by DCP and metformin as compared to dexamethasone control but these increases were not significant (Table 2).

## 4. Discussion

Phytochemical screening of DCP revealed the presence of compound** 1** that was assumed to be 8-(formyloxy)-8a-hydroxy-4a-methyldecahydro-2-naphthalenecarboxylic acid, since a derivative, namely, 8-formyl-7-hydroxy-5-isopropyl-2-methoxy-3-methyl-1,4-naphthaquinone, was isolated from the root bark [29] and the wood [30] of* Ceiba pentandra*. A glycosylated 3,4,5 trimethoxyphenol was isolated from the stem bark of* Bombax ceiba *[31, 32], a plant from the same family as* Ceiba pentandra*, suggesting that compound** 2** is a trimethoxyphenol. Compound 3 was identified as vavain, given that this compound has been previously isolated from* Ceiba pentandra* [33, 34].

Results from the present study show that daily administration of dexamethasone causes hyperglycemia, lipid dysregulation, increase in heart rate and mean arterial pressure, a drastic reduction of animals' relative body weight, and post-prandial glucose regulation. Some of these impairments such as hyperglycemia, lipid dysregulation, and postprandial glucose regulation were significantly corrected by DCP treatment.

It has been shown that treatment with dexamethasone results in muscle protein degradation [35, 36] and the inhibition of muscle protein synthesis [37], leading to skeletal muscle atrophy that may justify body weight loss. Concordantly in the present study, repeated administration of dexamethasone induced a marked reduction in tissue protein content. DCP tends to restore the protein content in the liver, heart, and aorta but its effect was significant only in the liver. More to that, DCP failed to prevent the body weight loss induced by dexamethasone. These results suggest that DCP is not able to efficiently interfere with the mechanism of skeletal muscle protein synthesis or loss. These results were somehow surprising given that one of the main paths leading to body weight loss is insulin resistance that potently suppress the IGF-1-dependent muscle protein synthesis [38, 39]. Results from the present study showed that DCP significantly and dose-dependently reduced the hyperglycemia induced by dexamethasone administration. Moreover, DCP also significantly and completely corrected glucose tolerance impairment induced by dexamethasone. Therefore, DCP could have corrected the body weight loss related to insulin resistance.

The rise in glycemia could result from the interaction of several dysfunctions. Indeed Makoto et al. [40] showed that dexamethasone decreased *β* cell sensitivity to glucose by reducing the level of GLUT2 which will therefore lead to a loss of the glucose regulation activity of pancreatic *β* cells. Jerrold and Olefsky [41] demonstrated that dexamethasone treatment markedly inhibited (by 50%; P < 0.05) both basal and insulin-stimulated glucose uptake in omental adipocyte. In addition, dexamethasone induces in the liver an increase in gluconeogenesis [42]. All these dysfunctions could have therefore led to an increase in insulin resistance/deficiency and elevate blood glucose. DCP and metformin administration inhibited the hyperglycemic effect of dexamethasone. These results suggest that DCP is able to prevent type II diabetes by either preventing insulin resistance and/or promoting insulin secretion. It has also been noticed that the extract was more effective in this context than metformin. This could be explained by the fact that metformin has no effect on insulin secretion but increases peripheral sensitivity to insulin [43], unlike extracts which might possess an insulinotropic activity. In addition, these results corroborate those of Dzeufiet et al. [6] and Olusola et al. [8] which showed that the stem and root bark of this plant have hypoglycemic effects in type I diabetic rats. More recently, Satyaprakash et al. [44] show that ethanol extract from the bark of* C. pentandra* was able to increase the serum insulin level in streptozotocin diabetic rats, therefore demonstrating the insulinotropic effect of the stem bark of the plant. Added to this, Fofie et al. [9] showed that DCP increases peripheral glucose consumption both in the liver and in skeletal muscles, further demonstrating the insulin-like effect of DCP.

Administration of various treatments induced a significant increase in the relative weight of the liver and the heart but the raw weight of these organs remained unchanged. These results indicate that the increase in relative organs' weight is related to the drastic body weight loss as observed. However, animals treated only with dexamethasone had a moderate left ventricular hypertrophy that was accompanied by elevated blood pressure. These irregularities might result from the reduction of NO level as shown by biochemical analysis of nitrites content. The present results corroborate those of Walker et al. [45] who showed that glucocorticoids inhibit the release of NO. Since NO, a potent endogenous vasorelaxant, has antimitogenic effect by inhibiting the growth and the proliferation of cardiovascular cells [46, 47], it could then justify the moderated elevated blood pressure and ventricular hypertrophy in dexamethasone-treated animals. The decoction of* C. pentandra* bark inhibited hypertension and hypertrophy induced by dexamethasone. Moreover, the extract has significantly increased the production of NO in the heart compared to the group treated only with dexamethasone. DCP could have seemingly counteracted the cardiovascular effect of dexamethasone by restoring the NO production and enhancing vascular relaxation and reducing cardiovascular tissues remodeling.

In addition to the mean arterial pressure and ventricular hypertrophy, dexamethasone increased the heart rate by 19.51%. These results are in accordance with those of Severino et al. [48], which have shown that animals treated with dexamethasone (0.1 mg/kg) had a heart rate increase of 4.55% as compared to control. Metformin induced a 14.97% decrease in heart rate of rats compared to the negative control. Contrariwise, extract administration was unable to reduce that tachycardia.

Dexamethasone had no influence on total cholesterol but significantly increased triglycerides level. These results are consistent with those obtained by Severino et al. [48] which have shown that dexamethasone is able to cause an increase in triglyceride levels by stimulating their production from the liver and elevating lipoprotein lipase activity in the adipose tissue. This elevated concentration of triglycerides has toxic effects at different levels. In *β*-pancreatic cells, accumulation of triglycerides is responsible for diminished expression of GLUT2 transporters, reduced secretion of insulin, and increased apoptosis stimulation [49]. The antidiabetic property of DCP can be explained by its lowering effect on triglycerides level, which will, therefore, ameliorate insulin secretion and insulin sensitivity.

Current knowledge about the linkage between oxidative stress and diabetes motivated the evaluation of some key oxidative stress parameters in the liver, the aorta, and the heart. It was found that glutathione was relatively stable in all organs except the liver. In this organ, dexamethasone depleted glutathione by 46.50% but the impairment was restored by extract administration. Glutathione through its antioxidant effects could have prevented the inhibition of IRS-1 autophosphorylation induced by stress-sensitive serine/threonine kinase which is able to inactivate the IRS-1 through a serine phosphorylation [50]. The restoration of hepatic glutathione by DCP may, therefore, promote glucose storage and increase glucose tolerance.

In the liver and the heart, dexamethasone had no effect on the level of catalase. But, the concentration of this antioxidant enzyme was increased after DCP administration. This could demonstrate that extract stimulates the* in vivo* synthesis of catalase.

## 5. Conclusion

Results from the present study showed that dried decoction from the stem bark of* C. pentandra* possesses potent antihyperglycemic effects and improves insulin resistance and dyslipidemia caused by dexamethasone. Seemingly, the antihyperglycemic effect of DCP results from its capacity of improving insulin resistance and potentiating peripheral glucose consumption. Its powerful antitriglyceride and antioxidant effects underline the importance of this plant extract in the treatment of type 2 diabetes.

## Figures and Tables

**Table 1:** Effect of repeated administration of the decoction of *Ceiba pentandra* (DCP) and metformin on the rats' plasmatic lipid content.

**Parameters (mg/dL)**	**Control**	**Dexamethasone (1mg/kg/day, *i.m*)**			
		**Distilled water**	**DCP 75**	**DCP 150**	**Metformin**
Total cholesterol	46.75 ± 3.77	46.27 ± 1.98	32.05 ± 7.52	31.72 ± 2.50*∗*^,a^	39.35 ± 4.27
HDL-cholesterol	16.19 ± 1.35	11.01 ± 1.44*∗*	12.06 ± 0.82*∗*	12.67 ± 1.25*∗*	12.24 ± 1.02*∗*
LDL-cholesterol	22.00 ± 4.51	17.40 ± 2.09	9.03 ± 1.74*∗*	6.32 ± 1.23*∗∗*	20.93 ± 3.42
Triglycerides	62.38 ± 8.99	104.70 ± 15.07*∗*	98.44 ± 12.26	69.33 ± 3.56*∗*^,†^	53.35 ± 6.42^†^

Values expressed as mean ± SEM; **∗**P<0.05 with respect to control group. ^†^P<0.05 with respect to dexamethasone group.

**Table 2:** Effect of repeated administration of the decoction of *Ceiba pentandra* (DCP) and metformin on catalase (CAT), superoxide dismutase (SOD), glutathione (GSH), malondialdehyde (MDA), and nitric oxide (NO) concentrations in the liver, heart, and aorta.

**Parameters assayed**	**Organs**	**Control**	**Dexamethasone (1 mg/kg/day, *i.m*)**			
			**Distilled water**	**DCP 75**	**DCP 150**	**Metformin**
**Proteins** (mg/g of tissue)	**Liver**	235,74 ± 19,15	187,10 ± 13,61*∗*	204,49 ± 9,40	271,31 ± 7,70^††^	257,05 ± 25,68^†^
	**Heart**	82,33 ± 3,88	71,25 ± 3,25	88,24 ± 10,27	83,86 ± 2,10	81,14 ± 3,12
	**Aorta**	33,91 ± 3,60	26,76 ± 1,67	27,86 ± 2,01	27,13 ± 1,98	33,91 ± 2,96

**Catalase** (activity/g of protein)	**Liver**	0.098 ± 0.014	0.135 ± 0.020	0.171 ± 0.024*∗*	0.150 ± 0.018	0.028 ± 0.007^††^
	**Heart**	0.446 ± 0.022	0.457 ± 0.011	0.954 ± 0.175^†^*∗*	0.874 ± 0.110	0.852 ± 0.142
	**Aorta**	8.378 ± 1.433	8.505 ± 0.741	8.679 ± 0.650	8.736 ± 0.846	7.215 ± 0.510

**SOD** (*μ*mol/g of protein)	**Liver**	0.036 ± 0.007	0.083 ± 0.024	0.043 ± 0.007	0.043 ± 0.011	0.035 ± 0.008
	**Heart**	0.173 ± 0.041	0.150 ± 0.020	0.151 ± 0.026	0.154 ± 0.047	0.187 ± 0.051
	**Aorta**	0.397 ± 0.118	0.680 ± 0.095	0.645 ± 0.089	0.704 ± 0.118	0.622 ± 0.030

**GSH** (*μ*mol/g of protein)	**Liver**	10,932 ± 0,397	7,37 ± 1,756*∗∗*	10,758 ± 2,34^†^	9,203 ± 2,052	8,777 ± 0,514
	**Heart**	9,316 ± 1,366	9,123 ± 2,000	8,964 ± 1,188	9,409 ± 3,857	9,416 ± 1,827
	**Aorta**	0,413 ± 0,056	0,486 ± 0,120	0,431 ± 0,050	0,405 ± 0,101	0,324 ± 0,034^†^

**MDA** (*μ*mol/g of tissue)	**Liver**	0.021 ± 0.001	0.020 ± 0.001	0.020 ± 0.002	0.017 ± 0.002	0.019 ± 0.001
	**Heart**	0.006 ± 0.001	0.007 ± 0.001	0.009 ± 0.001	0.009 ± 0.001	0.008 ± 0.001
	**Aorta**	0.001 ± 0.001	0.001 ± 0.003	0.004 ± 0.003	0.005 ± 0.001	0.003 ± 0.002

**NO** (*μ*mol/g of tissue)	**Liver**	0,535 ± 0,038	0,437 ± 0,032	0,574 ± 0,061	0,681 ± 0,107	1,208 ± 0,115^†††*∗∗*^
	**Heart**	0.098 ± 0.012	0.056 ± 0.006	0.096 ± 0.027	0.089 ± 0.012	0.083 ± 0.012

Values expressed as mean ± SEM; **∗**P<0.05; **∗∗**P<0.01, with respect to control group. ^†^P<0.05, ^††^P<0.01, ^†††^P<0.001, with respect to dexamethasone group.

## Data Availability

The data used to support the findings of this study are available from the corresponding author upon request.

## Abbreviations

*C. pentandra*: * Ceiba pentandra*
DCP: Decoction of the stem bark of* Ceiba pentandra*
SOD: Superoxide dismutase
T2DM: Type 2 diabetes mellitus
CVD: Cardiovascular diseases
GSH: Glutathione
MDA: Malondialdehyde.
